# Molecular identification and phylogenetic analysis of mitogenome of the *Xenocypris davidi* from Cao’e River

**DOI:** 10.1080/23802359.2019.1688099

**Published:** 2019-11-12

**Authors:** Haifang Xu, Yinjian Zhu, Daheng Zheng, Shoubao Yang

**Affiliations:** College of Life Sciences, Shaoxing University, Shaoxing, PR China

**Keywords:** *Xenocypris davidi*, mitochondrial genome, identification, phylogenetic analysis

## Abstract

In this study, the complete mitochondrial genome sequence of a *Xenocypris davidi* from Cao’e River was sequenced. The complete mitogenome of *X. davidi* was 16,630 bp in length, it contains the structure of 22 transfer RNA genes, 13 protein coding genes, 2 ribosomal RNA genes, and 1 non-coding region. The gene arrangement and organization in the mitogenome of *X. davidi* were in accordance with other Cyprinidae fishes. The results of phylogenetic analysis revealed that the mitochondrial genome sequence could provide useful information for the conservation genetics and evolution study of *X. davidi*.

*Xenocypris davidi* Bleeker, a medium-sized fish mainly distributed in the Changjiang River, Qiantang River, and Minjiang River of China (Xiao et al. 2001; Li et al. 2009; Xu et al. 2011; Tang et al. 2013). It feeds on epiphytic algae, underwater humus, and debris from higher aquatic plants, and therefore, it was cultured and used as an important water control fish in recent years (Xu et al. 2011; Zhang et al. 2016). The Cao’e River is one of the largest tributaries of the Qiantang River in Zhejiang province of east China. However, the wild population of *X. davidi* in Cao’e River decreases rapidly because of artificial irrigation works, environment pollution, and increasing capture pressure.

The mitochondrial genome sequence could be useful data in conservation genetics and evolution study for various aquatic species (Liu and Cui 2009; Chen et al. 2013; Yang et al. 2014; Wang et al. 2016) .

In this study, an individual of *X. davidi* was sampled from the Cao’e River (29°57′52.7′′N 120°52′32.4′′E), its complete mitogenome was sequenced and identified (designated *XD-1*). The previously published mitogenome of *X. davidi* (GenBank accession no. KF039718, designated *XD-2*) was used to design PCR amplification primers (Liu 2014). The *XD-1* specimen and its DNA are kept in the Shaoxing Aquatic Service Platform now.

The complete mitochondrial genome of *X. davidi* is 16,630 bp in length (GenBank accession no. MN264265), it contains 22 tRNA genes, 13 protein-coding genes (PCDs), 2 rRNA genes, and one non-coding region. These genes showed a conserved arrangement with other Cyprinidae fishes (Yang et al. 2014; Jia et al. 2016).

The total length of the PCD sequences is 11,425 bp. The total length of all tRNA genes was 1566 bp, varying from 68 bp (tRNA^Cys^) to 76 bp (tRNA^Leu^ and tRNA^Lys^). The 12S rRNA gene (961 bp) and 16S rRNA (1693 bp) gene of *X. davidi* are located between the tRNA^Phe^ and tRNA^Leu(UUR)^gene, and are separated by the tRNA^Val^ gene. The D-loop region is 935 bp in length, which is located between the tRNA^Pro^ and tRNA^Phe^ gene. All of the PCD genes except *COX I* (started with a GTG) are initiate with an ATG. Five PCD genes terminate with TAA (*ND1*, *COXI*, *ATP6*, *ND4L*, and *ND6*), three PCDs (*COX II*, *ND3*, and *Cyt b*) ended with T–, two PCDs (*COX III* and *ND4*) ended with TA-, whereas three PCDs (*ND2*, *ND5*, and *ATP8*) ended with TAG (data not shown). The overall nucleotide composition of *X. davidi* mitogenome is A: 31.24%, G: 16.19%, T: 25.38%, and C: 27.18%, respectively. Which showed an similar A + T rich feature (56.62%) with other vertebrate mitogenomes (Tzeng et al. 1992; Jondeung et al. 2007; He et al. 2014).

The generated phylogenetic tree indicated that *X. davidi* is clustered in genus Xenocypris as expected (Figure 1), but it showed distant kinship with other Cyprinidae fishes. This results indicated that the mitogenome sequence could be useful information in the field of conservation genetics and evolution for aquatic species.

**Figure 1.**
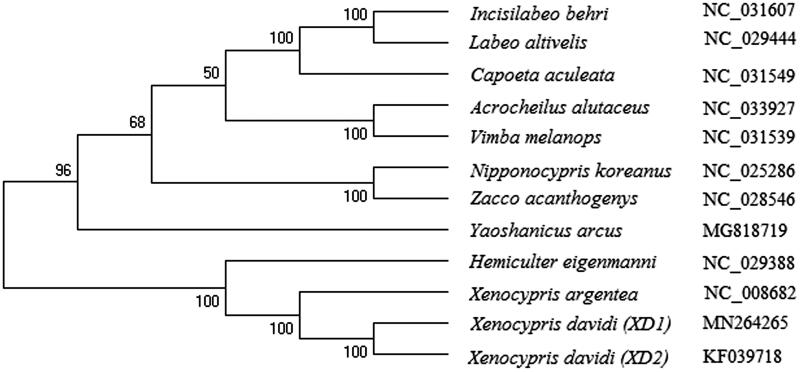
The phylogenetic analysis of *X. davidi* and other Cyprinidae fishes based on the mitogenome sequences.
